# The Immobilization Mechanism of Inorganic Amendments on Cu and Cd in Polluted Paddy Soil in Short/Long Term

**DOI:** 10.3390/toxics12020157

**Published:** 2024-02-17

**Authors:** Qing Liu, Yuan Ding, Yuqi Lai, Yan Long, Hong Shi, Min Liu

**Affiliations:** 1 College of Environment and Chemical Engineering, Nanchang Hangkong University, Nanchang 330063, China; lqing77a@163.com (Q.L.); hellolyq123@163.com (Y.L.); atang98@163.com (Y.L.); 2 National-Local Joint Engineering Research Center of Heavy Metals Pollutants Control and Resource Utilization, Nanchang Hangkong University, Nanchang 330063, China; 8659979@163.com; 3 Jiangxi Key Laboratory of Agricultural Efficient Water-Saving and Non-Point Source Pollution Preventing, Jiangxi Central Station of Irrigation Experiment, Nanchang 330063, China; 4 Jiangxi Ecological Environment Monitoring Center, Nanchang 330039, China

**Keywords:** copper, cadmium, iron oxides, aging mechanism, activation risk

## Abstract

This study investigated the impact of soil colloidal characteristics on the transfer patterns of different Cu and Cd speciation in contaminated soil treated with three different amendments: lime (L), zero-valent iron (ZVI), and attapulgite (ATP). It seeks to clarify the activation hazards and aging processes of these modifications on Cu and Cd. Compared with the control (CK), the available Cu concentrations treated with amendments reduced in the short term (6 months) by 96.49%, 5.54%, and 89.78%, respectively, and Cd declined by 55.43%, 32.31%, and 93.80%, respectively. Over a 12-year period, there was no significant change in the immobile effect with L, while Cu and Cd fell by 19.06% and 40.65% with ZVI and by 7.63% and 40.78% with ATP. Short- and long-term increases in the readily reducible iron and manganese oxide fraction of Cu and Cd were accompanied by a considerable rise in the concentrations of amorphous iron oxide in the soil and colloid after amendment treatment. This suggested that Cu and Cd were immobilized and stabilized in part by amorphous iron oxide.

## 1. Introduction

South China is well-known for its wealth of mineral resources, which results in high levels of heavy metal concentration in the environment. In these areas, acidification and heavy metal contamination often coexisted. As illustrated by Ding et al. [1], in the vicinity of the copper smelting facility in Jiangxi Province, the soil pH varies between 4.04 and 5.16, while the concentrations of Cu and Cd in the soil are 1294.63 mg/kg and 9.06 mg/kg, respectively. Therefore, in such circumstances, vegetation finds it difficult to flourish. Improving the environmental quality of the soil and restoring the production and service function of contaminated agricultural land are vital steps.

Alkaline materials, clay minerals, metals and their oxides, and other inorganic compounds have been used to immobilize Cu and Cd in contaminated soil. This technique has become essential for soil remediation, especially when combined with the physicochemical properties of the soil in southern China. It is well known that by increasing the pH of acidic soil, lime (L) may lower the available Cu and Cd concentrations in soil [2]. When soil acidity and redox conditions vary, zero-valent iron (ZVI) could transform into several types of iron oxides. By means of adsorption, complexation, and precipitation, these oxides may interact with soil heavy metals and hence decrease their availability [3]. Attapulgite (ATP), a water-bearing magnesium-aluminosilicate clay mineral with a layered chain structure and a large specific surface area, demonstrates strong adsorption capacity and excellent thermal stability, which can reduce the concentrations and activities of heavy metal ions in soil by adsorption, coprecipitation, complexation, and coordination [4].

Long-term alterations in the external environment, however, could allow the heavy metals that the amendments neutralized to become active afterwards. This explains why there has been a great deal of debate over the immobilization of soil heavy metals via additives. Consequently, there has been a lot of attention in the sector on the immobility impact of modifications. Wu et al. [5] applied a composite amendment comprising limestone and zeolite to remediate Cd and Pb-contaminated soil for a duration of 3 years. The findings revealed that within the first year, the soil pH experienced an increase of 1.95, while the exchangeable Cd content reduced by 98.9% when the dosage reached 8 g/kg. Over time, the amendment’s immobilization effect persisted, with the exchangeable Cd content remaining 71.4% lower than that of the control group. In-situ remediation experiments done by Cui et al. [6] around a copper smelter over the course of five years showed that adding lime caused the pH to rise by 1.30 within six months and the concentrations of available Cu and Cd decreased by 23% and 5.3%. However, the soil pH and the immobile effects gradually decreased as time passed by. Several studies have used simulation methods, such as high-temperature experiments [7] and column tests, to simulate the aging process. Hartley et al. [8] conducted leaching experiments to study the effects of goethite (α-FeOOH), iron grit, iron (II) and (III) sulfates (plus lime), and other iron oxides on the activity of As in soil, and used column tests to simulate long-term immobile effects. Studies have shown that clay minerals have a good immobile effect on heavy metals in soil [9], but stability research on clay minerals on heavy metals is quite scarce. The aging effects of the amendments aforementioned on heavy metals deserve more study, although all of them have been proven effective in reducing the availability of heavy metals by neutralization, adsorption, complexation, and re-oxidation.

Iron oxides are important secondary minerals in tropical and subtropical soils, characterized by a large specific surface area and abundant surface hydroxyl groups. They are the main components responsible for the adsorption and immobilization of heavy metals in soil [10]. Research has shown that environmental factors, such as pH, can affect the species and structure of iron oxides, which can change the adsorption capacity and mechanisms of heavy metals by submicrostructure [11,12]. Other studies have shown that pH in acidic paddy soils in the southern regions is an important factor affecting the Cd concentration in rice grains. Also, the Fe concentration in amorphous iron oxide has a significant impact on the Cd concentration in grains [13]. The interaction between soil iron oxides and heavy metals is crucial for evaluating the immobile effect and aging mechanism of amendments. Unfortunately, early studies did not consider the speciation changes of in-situ iron oxides in soil and their impact on the transfer patterns of Cu and Cd in soil after the application of three amendments, namely L, ZVI, and ATP. It is well-known that colloids are the smallest and most active component in soil. The abundant iron oxides present in colloids provide an effective approach and method to explore the immobile effects and mechanisms of the amendments on heavy metals in soil [14].

The indoor incubation experiment is easy to conduct, and the experimental results are generally similar to the plot experiment, so it is often used instead of the plot experiment. He et al. [15] conducted indoor incubation studies and in-situ plot tests concurrently for a 4-month period using calcium magnesium silicon fertilizer and organic fertilizer as amendments. The findings verified that under both incubation settings, the exchangeable Cd dropped by 11.2% and the pH of the soil rose by 18.4% and 15.2%, respectively. The study showed that the short-term effects of indoor incubation studies are the same as those of plot experiments. Thus, long-term positioning pot studies’ early design flaws may be efficiently corrected by indoor incubation experiments, which can also aid in investigating the process of soil heavy metal immobilization.

Thus, in order to address the following issues: (1) the immobile effects and differences of the three amendments on Cu and Cd in short/long term incubation; (2) elucidating the aging mechanisms of amendments on Cu and Cd and the activation risks of heavy metals, L, ZVI, and ATP were chosen as the tested amendments to immobilize Cu and Cd in polluted soil in short-term indoor incubation (6 months) and long-term plot (12 years) incubation.

## 2. Materials and Methods

### 2.1. Test Material and Experimental Design

**Figure 1:**
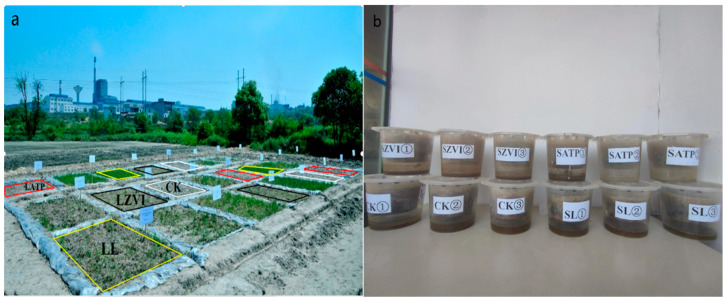
(**a**) Long-term plot incubation; (**b**) short-term indoor incubation.

The soil samples were collected from a paddy field near a smelter in Jiangxi Province, China. Lime (L), zero-valent iron (ZVI), and attapulgite (ATP) treatments were carried out in triplicate for indoor/plot incubation to study the immobile effect and aging mechanisms of Cu and Cd in polluted soil. The specific plan was as follows:

Long-term plot incubation experiment (12 years): The experiments were designed with the land split into randomised blocks or plots. Each plot was 6 m^2^ (3 m × 2 m) and the plots were separated by plastic plates. The treatments applied were (1) 0.21% L (*w*/*w* according to the mass of surface 20 cm soil), (2) 1.16% ZVI, (3) 2% ATP, and (4) the control (CK). (Figure 1a), labeled LL, LZVI, and LATP [16,17]. Ryegrass was sown twice in December 2008 and December 2010, and the last harvest was in July 2011. During ryegrass planting, there was no impact on the experimental site. Before sampling in June 2020, no amendments were applied in each treatment, and normal management was still maintained. Surface soil samples (0–20 cm) of each treatment were collected using a diagonal sampling method and air-dried for use.

Short-term indoor incubation experiment (6 months): The test soil was gathered from the contaminated location that was noted in the long-term pot experiment (Table 1 lists the basic physicochemical characteristics of the soil). The particular method used for the experiment was as follows: As seen in Figure 1b, three different types of amendments—L, ZVI, and ATP—were individually incorporated into the CK soil at the same dose as the plot experiment and labeled SL, SZVI, and SATP, respectively. Furthermore, throughout the incubation phase, the soil’s water content was maintained at 65% of its water-holding capacity. The short-term effects and mechanisms of amendments on contaminated soil were studied after six months of incubation [18].

### 2.2. Sample Analysis/Experimental Methods

Extraction of soil colloids: Extraction of soil clay colloids less than 2 μm by sedimentation + siphon method [19]

Speciations analysis of iron in soil and colloid: The speciations of free iron in soil and colloid can be divided into amorphous iron oxide and crystalline iron oxide, with specific methods detailed in reference [20].

The physicochemical properties of the soil were analyzed according to the method of Bao [20]. The soil pH was measured using a pH meter (PHS-3C, Shanghai, China) at a water-to-soil ratio of 2.5:1 (*v*/*m*). Soil organic matter (SOM) was determined using the K_2_Cr_2_O_7_. The total concentrations of Cu, Cd, and Fe in the soil/colloid samples were determined using an inductively coupled plasma mass spectrometer (ICP-MS, Agilent 8900, Palo Alto, CA, USA) after digestion with HCl–HNO_3_–HClO_4_.

Analysis of available Cu and Cd concentrations in soil: 0.01 mol·L^−1^ CaCl_2_ was used as the extractant to extract the concentrations of Cu and Cd in the soil, which were then determined by inductively coupled plasma mass spectrometer (ICP-MS, Agilent 8900, Palo Alto, CA, USA) to characterize the available concentrations of heavy metals in the soil [21].

Speciations analysis of Cu and Cd in soil: To analyze the distribution of Cu and Cd speciation in soil and investigate the effects of soil iron oxides on the transfer of Cu and Cd, varying concentrations of Cu and Cd in the soil samples were sequentially extracted using a modified Tessier method. This method allowed for the identification of Cu and Cd speciation in the exchangeable fraction, carbonate fraction, readily reducible iron and manganese oxide fraction, organic fraction, crystalline iron oxide fraction, and residual fraction [22].

Speciations analysis of Cu and Cd in soil colloid: Heavy metal speciations in soil colloid can be classified as an amorphous iron oxide fraction, a crystalline iron oxide fraction, and a residue fraction [22].

Morphology analysis of soil colloid iron oxides: the colloid samples can first be treated with oxalate-oxalate ammonium oscillation in the absence of light to remove amorphous iron oxide [23], and then the deamorphized colloid samples can be pressed into powder pellets for X-ray diffraction analysis technique (XRD, D8 Advance X-ray diffractometer; Bruker, Karlsruhe, Germany).

### 2.3. Data Analysis

Data processing and graphing were done, using Microsoft Excel 2010 and Origin 8.5 software, while statistical analysis can be performed using SPSS 18.0 software. Duncan’s multiple range test was performed to determine the difference between treatments. Means were compared using the least significant difference test at a *p* < 0.05 level of significance, and letters were used to suggest the significant variations in the data set. All of the results in the tables and figures were described as means ± standard deviation (*n* = 3).

## 3. Results and Discussion

### 3.1. Soil

#### 3.1.1. The Variation of Soil pH in Indoor/Plot Incubation

According to Cui et al. [16], the contaminated soil in the experimental region had a pH of 4.54 in 2009 (data from the literature; no significant analysis was done). When we resampled and reanalyzed the test soil in 2020, the results indicated a drop of 0.31, with a pH of 4.23. The following are the primary causes of this decline: First off, the research location is situated in China’s acid deposition area, which includes the middle and lower branches of the Yangtze River. Long-term acid deposition has caused a drop in soil pH in this area [24]. Second, there’s a chance that the two sample periods’ sampling conditions—season, temperature, humidity, etc.—were not totally constant, which affected the experimental findings. Therefore, the contaminated soil collected in 2020 was utilised as the control soil (CK) in this work to analyse the immobile effects and mechanisms of L, ZVI, and ATP in order to maintain uniformity of analytical conditions.

**Figure 2:**
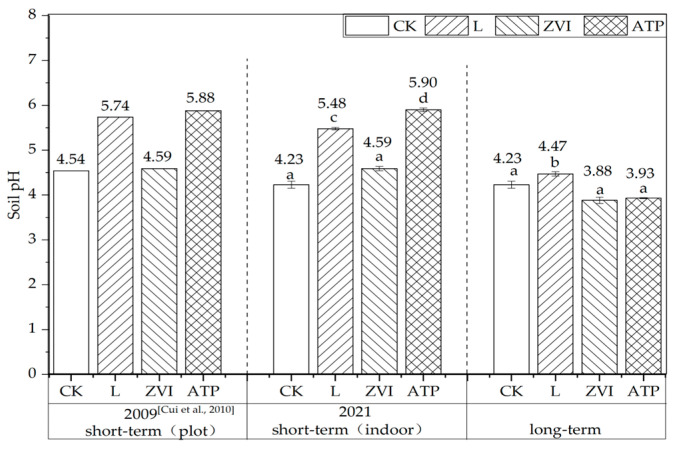
Soil pH before and after three kinds of inorganic amendment treatment in short/long term [16]. Note: Different letters indicated significant differences between different treatments (*p* < 0.05) in the same incubation. Consider this indication for the following graphs.

The variations of soil pH in short-term (6 months) indoor/plot incubation are shown in Figure 2. The soil pH treated with L, ZVI, and ATP increased by 1.20, 0.05, and 1.34 respectively in plot incubation, compared with CK (4.54). Similarly, the soil pH increased by 1.25, 0.36, and 1.67 respectively in indoor incubation, compared with CK (4.23). The changing patterns of pH in plot incubation were consistent with indoor incubation, indicating similar effects between indoor and plot incubation experiments in short-term. Therefore, the plot incubation experiment can be substituted by an indoor incubation experiment to study the effect of amendments on polluted soil in short-term.

Following the administration of L, ZVI, and ATP, the pH value increased in the short term from 4.23 (CK) to 5.48, 4.59, and 5.90, respectively. In addition, after 12 years, the value from 4.23 (CK) to 4.47, 3.88, and 3.93. When comparing the pH value of long-term treatment with ZVI and ATP to that of CK, no significant differences were seen. Furthermore, even though there was a substantial difference between the groups treated with L and CK, the absolute value was only 0.24.

#### 3.1.2. Immobile Effects of Three Amendments on Soil Cu and Cd in Short/Long Term Incubation

According to Yang et al. [25], the most active heavy metals in soil are extracted with CaCl_2_. In addition, the extraction of heavy metals with CaCl_2_ has been advocated by the “Technical Regulations for Soil Sample Analysis and Testing Methods for the Detailed Investigation of Soil Pollution in China”. The present paper examines the immobile effects of three inorganic amendments on soil heavy metals using CaCl_2_-extracted Cu and Cd (Labeled as CaCl_2_-Cu and CaCl_2_-Cd) [26].

**Figure 3:**
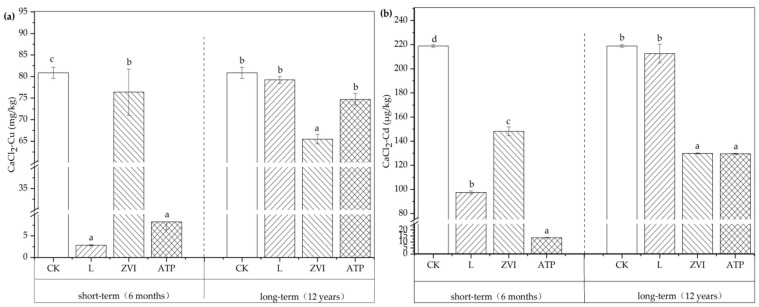
(**a**) Variations of available Cu concentration in soil; (**b**) Variations of available Cd concentration in soil. Note: Different letters indicated significant differences between different treatments (*p* < 0.05) in the same incubation.

The variations of available Cu and Cd concentrations in soil are shown in Figure 3. In short-term incubation, compared with CK, the CaCl_2_-Cu concentrations decreased by 96.49%, 5.54%, and 89.78%; and the CaCl_2_-Cd concentrations by 55.43%, 32.31%, and 93.80% respectively with the application of the L, ZVI, and ATP. The results indicated that the amendments can significantly reduce the available Cu and Cd concentrations in soil. The immobile effect on Cu is L > ATP > ZVI in turn, while Cd is ATP > L > ZVI.

The changes in CaCl_2_-Cu and CaCl_2_-Cd showed different patterns after the application of L, ZVI, and ATP for 12 years of aging. Compared with CK, the reductions of amendments were 2.02%, 19.06%*, and 7.63% for Cu, and 2.83%, 40.65%*, and 40.78%* for Cd, respectively (the value with * indicates *p* < 0.05). Among them, the immobile and stable effect of ZVI is the most exquisite. This finding aligns with the study conducted by Danila et al. [7], which demonstrated Cu no solo may be stabilized over an extended period of time via thermal ageing experiments using zero-valent iron. When ATP and CK were tested, the immobile impact on Cd remained considerable and showed concordance with ZVI. However, Cu gradually decreases and does not demonstrate any longer significant changes over time. Consistent with our findings, Zhang et al.’s [27] experimental results shown that palygorskite’s immobile impact on soil Cd, Cu, Pb, and Zn may be maintained for six years, with 0.5 to 1 year being the best effect. After long-term incubation with L, there were no significant differences in the available concentrations of Cu and Cd between L and CK, which were consistent with the findings of Cui et al. [28,29]. Their study showed that the effects of lime (at a dosage of 0.2% on Cu and Cd) weakened over time and there were no significant differences in the available concentrations of Cu and Cd compared to CK after 7 years, although they were significantly lower within 3 years.

#### 3.1.3. Variations of the Extraction Fraction of Soil Cu and Cd with Three Amendments in Short/Long Term Incubation

A modified sequential extraction method based on the traditional Tessier five-step sequential extraction method was selected in this paper [30]. The speciation fractions of soil Cu and Cd were divided into the exchangeable fraction, the carbonate fraction, the readily reducible iron and manganese oxide fraction, the organic fraction, the crystalline iron oxide fraction, and the residual fraction. So, readily reducible iron and manganese oxide fractions and crystalline iron oxide fractions on the distribution and potential activity of heavy metals will be emphasized in this paper.

**Figure 4:**
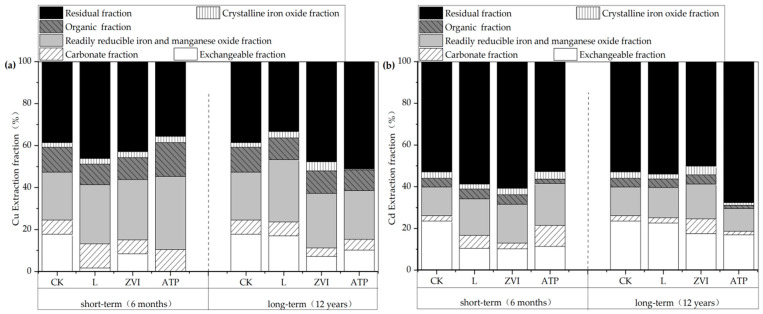
(**a**) The fraction distribution of soil Cu in short/long term; (**b**) The fraction distribution of soil Cd in short/long term.

The variations of speciations of soil Cu are shown in Figure 4a. In short-term incubation, compared with the CK, the soil Cu fractions treated with L were transformed from an exchangeable fraction (−16.07%) to a carbonate fraction (+4.74%), a readily reducible iron and manganese oxide fraction (+5.43%), and a residual fraction (+7.62%). The soil Cu fractions treated with ZVI were transformed from an exchangeable fraction (−9.30%) to a readily reducible iron and manganese oxide fraction (+6.00%), a crystalline iron oxide fraction (+0.59%), and a residual fraction (+4.33%). The soil Cu fractions treated with ATP were transformed from an exchangeable fraction (−17.75%) to a carbonate fraction (+3.64%), a readily reducible iron and manganese oxide fraction (+12.15%), an organic fraction (+4.23%), and a crystalline iron oxide fraction (+0.70%). This indicated Cu no solo was transformed from the easily absorbable speciations to the potentially available and unavailable speciations under the treatment of the three amendments. At a longer time scale, the fraction of soil Cu treated with L showed the transformation from a residual fraction (−5.12%) to a readily reducible iron and manganese oxide fraction (+7.05%) and a crystalline iron oxide fraction (+0.81%). The fraction of soil Cu treated with ZVI showed the transformation from an exchangeable fraction (−10.51%), a carbonate fraction (−2.86%), and a readily reducible iron and manganese oxide fraction (−3.15%) to a crystalline iron oxide fraction (+2.11%) and a residual fraction (+15.60%). The fraction of soil Cu treated with ATP showed the transformation from an exchangeable fraction (−7.57%) to a residual fraction (+12.56%). In contrast to those treated with L and ZVI, the transfer patterns over the long term of soil Cu speciations after being treated with ATP showed significant changes, indicating a different immobilization mechanism of ATP on soil Cu.

The variations of speciations of soil Cd are shown in Figure 4b. Under the treatment of the three amendments, the transfer patterns of speciations of soil Cd were generally consistent with Cu.

### 3.2. Soil/Colloid

#### 3.2.1. Variations of In-Situ Iron Speciations in Soil/Colloid with Three Amendments in Short/Long Term Incubation

**Figure 5:**
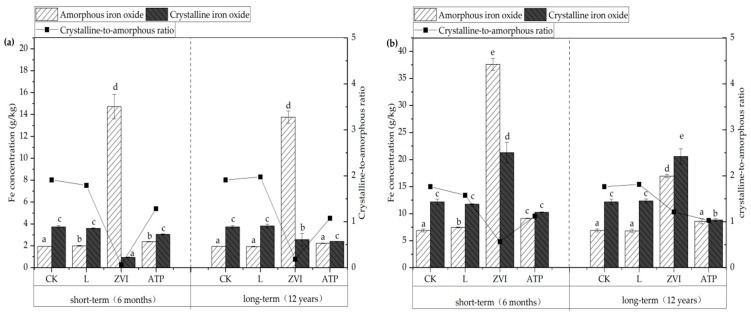
(**a**) The fraction distribution of Fe in soil; (**b**) The fraction distribution of Fe in soil colloid. Note: Different letters indicated significant differences between different treatments (*p* < 0.05) in the same incubation.

Iron oxides are important parts of soil and colloid. Moreover, transfer mechanisms of Cu and Cd mainly depended on variations in the speciations and crystalline of iron oxides in soil/colloid in short/long term incubation (as shown in Figure 5).

The variations of speciations of soil Fe are shown in Figure 5a. After L, ZVI, and ATP treatments during short-term incubation, the soil’s amorphous iron oxide concentration rose by 2.72%, 653.70%, and 21.76%, respectively, in comparison to the CK. Compared to the CK, which received ZVI treatment and shown the greatest variance, these variations were considerably different. According to Bae et al. [31], ZVI is very reactive and readily experiences redox reactions in soil, which could result in the transformation into amorphous iron oxide. Following treatment with L, ZVI, and ATP, there were notable variations in the concentrations of amorphous iron oxide after a prolonged incubation period. The discrepancies of amorphous iron oxide concentration L and ATP groups progressively diminished and became non-significant compared with the CK. However, after being treated with ZVI, the amorphous iron oxide concentration remained much greater than the CK. The study by Kumpiene et al. [32] also shown that the soil continued to retain a significant proportion of metastable and highly active amorphous/weakly crystalline iron oxides even after 15 years of ZVI treatment. The Fe speciations in the soil colloid are shown in Figure 5b, and with the addition of L, ZVI, and ATP, the pattern of change in the soil’s iron speciations is comparable to that of the soil.

The variations in the crystalline-to-amorphous ratios of soil and colloid after applying the L, ZVI, and ATP are generally consistent and significantly lower than the CK, according to further analysis of the crystalline-to-amorphous ratio (the ratio of crystalline iron oxide to amorphous iron oxide concentration) conducted during short-term incubation. The variations in crystalline-to-amorphous ratios between those treated with L and CK in both soil and colloid become insignificant as time passes, but the differences between those treated with ZVI, ATP, and CK remain substantial. Rashid et al. [33] found that there is a strong correlation between pH and variations in soil iron oxide concentration. In particular, the crystalline-to-amorphous ratio rises when the soil reacidifies. This result is in line with the long-term impacts of L treatment in the soil.

#### 3.2.2. Aging Trends of Cu and Cd in Various Speciations in Soil/Colloid

The total concentration of Cu and Cd in the compared soil colloid were 1509.99 mg·kg^−1^ and 869.71 μg·kg^−1^, respectively, which were much higher than the total concentration of Cu and Cd in the soil (as described in the Materials and Methods section). This is primarily because soil colloids are the finest, most active component of the soil with a large surface area, and possess strong capabilities for absorbing and retaining heavy metals [34].

Following treatment with L, ZVI, and ATP, the Cu and Cd concentration in the colloid was considerably greater than that in the CK during short-term incubation (Table 2). The patterns of Cu and Cd concentration in colloid after treatment with L, ZVI, and ATP, however, were not consistent with those seen in the short-term during long-term incubation. Following application of the L treatment, there were no discernible variations in the Cu and Cd concentrations of colloids when compared to the CK. Cu and Cd concentration in the colloid remained much higher than the CK after ZVI therapy. Following ATP treatment, there was a substantial change in the Cd concentration of the colloid compared to the CK, but not in the Cu concentration. The concentration of heavy metals in colloids is positively connected with the immobile impact on Cu and Cd in the soil, according to the changes in the available Cu and Cd concentration in soils treated with the three amendments.

**Figure 6:**
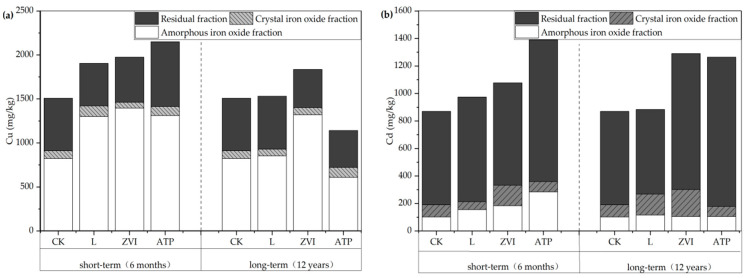
(**a**) The fraction concentration of soil colloid Cu in short/long term; (**b**) The fraction concentration of soil colloid Cd in short/long term.

Further analysis of the distribution of Cu and Cd speciations in soil colloids after the application of three amendments at different time scales is shown in Figure 6a. In short-term incubation, the amorphous iron oxide fraction of Cu in colloid after being treated with L, ZVI, and ATP increased by 13.77%, 16.14%, and 6.44%, respectively. However, in long-term incubation, the amorphous iron oxide fraction of Cu in colloid showed different patterns after the application of the amendments, an increase of 1.14%, an increase of 17.33%, and a decrease of 1.22%. This variation patterns is consistent with the changes in the total Cu concentration in the colloid. Based on the results of the analysis of available concentration and speciations transformation of heavy metals in the soil, the amorphous iron oxide fraction of Cu in colloid can intuitively reflect the immobile effect of the three inorganic amendments in short/long term incubation. This indicated that the amorphous iron oxide fraction of heavy metals in colloid is an effective pathway for immobilization, and it also suggests that changes in the concentration of amorphous iron oxide in colloid may induce the reactivation of heavy metals in the soil.

Figure 6b displays the different speciations of colloid Cd. The transition patterns of Cd speciations in colloids is comparable to that of Cu after applying L and ATP treatments. In the short-term incubation period after ZVI treatment, the amorphous iron oxide fraction of Cd in the colloid increased in comparison to the CK. On the other hand, after the long-term incubation, Cd in the colloid changed from amorphous iron oxide bound to crystal iron oxide bound. The amorphous iron oxide fraction of Cd dropped by 3.37%, whereas the crystal iron oxide fraction of Cd rose by 5.39% when compared to the CK. The crystalline phase transformation of iron oxide among different heavy metal ions is related to their redox potentials [35], and the principles guiding the promotion of crystalline phase transformation in ZVI by various heavy metals can differ [36,37,38]. Additionally, this shows that there are still two avenues for the amorphous iron oxide bond fraction to re-emerge and turn into the crystal iron oxide bound fraction over an extended period of time, suggesting that it is a transitional speciation in the stabilization of heavy metals.

#### 3.2.3. Impact of Soil Colloid Characteristics on the Transfer Trends of Soil Cu and Cd in Short/Long Term Incubation

**Figure 7:**
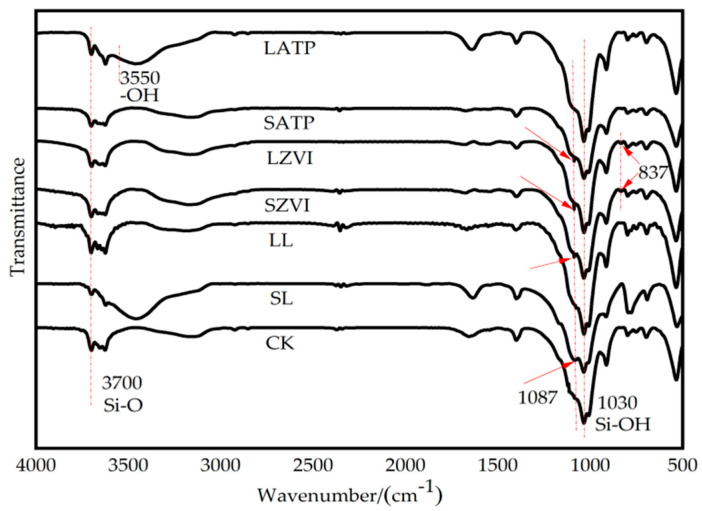
FTIR spectra of soil colloid treated with three kinds of amendments in short/long term.

Using fourier transform infrared spectroscopy (FTIR, Vertex 70, Bruker, Karlsruhe, Germany), we examined the changes in the functional groups of the colloid after Cu and Cd adsorption by three amendments in the short and long term. The structure of the colloid was not disrupted by the addition of L, ZVI, and ATP, as demonstrated by the lack of significant changes in the infrared spectral characteristic peaks of the colloid in the CK and those treated with the three different amendments, as shown in Figure 7.

The amorphous iron oxide (goethite) exhibited characteristic absorption peaks around 1087 cm^−1^ [39] in short-term treatment (SL, SZVI, and SATP). Over time, the intensity of this absorption peak decreased significantly for LL and LATP, demonstrating no significant difference with CK; in the meantime, the effect of L treatment on heavy metals displays no significant difference with CK. The results indicated the involvement of amorphous iron oxide in the reaction in the short term [40]. Due to its large specific surface area and high adsorption capacity, amorphous iron oxide can react with dissolved metals to reduce their mobility [7], thereby playing a role in the immobilization of heavy metals in colloids. Meanwhile, although the intensity of the hydroxide absorption peak in LATP weakened, the -OH functional groups located at 3550 cm^−1^ on the surface of ATP were still able to coordinate and bind with heavy metals, enhancing the long-term transformation of heavy metals from the reduced and acidic soluble fraction to the oxidized and residual fraction [41]. In long-term incubation, ZVI still exhibited the characteristic iron oxide hydroxide peak at 1087 cm^−1^. Additionally, the Fe-O characteristic peak of ZVI itself at 837 cm^−1^ formed Fe-O-Cu and Fe-O-Cd complexes with Cu and Cd in the soil, contributing to the long-term effectiveness of ZVI in the immobilization of heavy metals.

**Figure 8:**
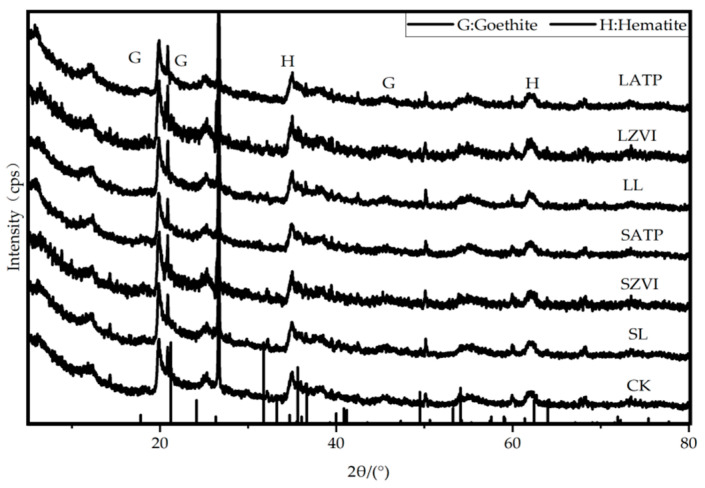
XRD patterns of soil colloid with the removal of amorphous iron oxide in short/long term.

The X-ray diffraction (XRD, D8 Advance X-ray diffractometer; Bruker, Karlsruhe, Germany) spectra of colloid without amorphous iron oxide (Figure 8) was analyzed. It revealed that in short/long term incubation treated with the three inorganic amendments, four sharp peaks can be observed around 2θ of 20°, 45°, 35°, and 62°, which correspond to the characteristic peaks of goethite and hematite in the reference card standard spectra. It can be observed that there is no significant change in the types of crystalline iron oxide in colloids after treatment with the three amendments in short/long term incubation. Additionally, no new peaks were observed, indicating that the transformation of goethite and hematite fraction of Cu and Cd in long-term immobile process after being treated with L, ZVI, and ATP did not exhibit significant variations.

## 4. Conclusions

(1) Compared to CK, L, ZVI, and ATP could dramatically lower the available Cu and Cd concentrations in a short period of time (6 months). The available Cu concentrations declined by 96.49%, 5.54%, and 89.78%, respectively, while the available Cd concentrations decreased by 55.43%, 32.31%, and 93.80%, respectively. L > ATP > ZVI was the immobile impact on Cu among them, while ATP > L > ZVI was the immobile effect on Cd. ZVI and ATP continue to have strong immobile effects on Cu and Cd over a 12-year duration, although L has no comparable effects.

(2) The immobile and stable effects of Cu and Cd are directly impacted by changes in the concentration of amorphous iron oxide in soil and colloids. The concentration of amorphous iron oxide rose considerably in short-term incubation after the application of L, ZVI, and ATP, with increases of 2.72%, 653.70%, and 21.76%, respectively, compared with the CK. This resulted in a reduction in the activity of Cu and Cd in soil. The amorphous iron oxide change patterns brought on by the amendments in long-term incubation were quite distinct. Among these, the concentration of amorphous iron oxide is still much greater than that of CK that has had ZVI and ATP treatment. The concentrations of Cu and Cd that are available then drop accordingly. On the other hand, after L treatment, the available Cu and Cd in soil could be increased.

(3) It has been proven that iron oxides in soils/colloids can play a role in immobilizing heavy metals by altering their binding speciations with heavy metals. However, the transformation of iron oxides during the process of immobilizing heavy metals, as well as the factors influencing this transformation, are worth further exploration. This has significant implications for the long-term stabilization of heavy metals.

## Figures and Tables

**Table 1:** Basic physical and chemical properties of the tested soil.

	pH	SOM/%	Total Cu/mg·kg^−1^	Total Cd/mg·kg^−1^	Total Fe/g·kg^−1^
Soil	4.23 ± 0.07	2.70 ± 0.09	664.31 ± 9.72	0.91 ± 0.03	19.14 ± 0.57

Note: The values were described as means ± standard deviation (*n* = 3).

**Table 2:** Effects of three kinds of inorganic amendments on Cu and Cd concentration of the soil colloid in short/long term.

	Short-Term		Long-Term	
	Cu/mg·kg^−1^	Cd/μg·kg^−1^	Cu/mg·kg^−1^	Cd/μg·kg^−1^
CK	/	/	1509.99 ± 48.61 ^a^	869.71 ± 7.86 ^a^
L	1904.21 ± 24.41 ^b^	973.49 ± 18.08 ^b^	1561.00 ± 80.78 ^a^	883.72 ± 12.37 ^a^
ZVI	1974.96 ± 37.25 ^c^	1077.73 ± 91.02 ^b^	1835.89 ± 18.08 ^b^	1260.23 ± 51.69 ^c^
ATP	2150.36 ± 10.18 ^b^	1390.02 ± 20.05 ^c^	1142.77 ± 7.74 ^a^	1263.65 ± 15.27 ^b^

Note: The CK data were measured in 2021. Different letters indicated significant differences between the same treatments (*p* < 0.05) in the different periods.

## Data Availability

The authors declare that data supporting the findings of this study are available within the article.
